# Complete Genome Sequence of an Adenovirus-1 Isolate from an African Pygmy Hedgehog (*Atelerix albiventris*) Exhibiting Respiratory Symptoms in Japan

**DOI:** 10.1128/MRA.00695-19

**Published:** 2019-10-03

**Authors:** Hiroo Madarame, Jumpei Uchiyama, Kenichi Tamukai, Yukie Katayama, Nanako Osawa, Kaoru Suzuki, Tetsuya Mizutani, Hideharu Ochiai

**Affiliations:** a Veterinary Teaching Hospital, Azabu University, Sagamihara, Kanagawa, Japan; b Laboratory of Veterinary Microbiology I, Department of Veterinary Medicine, Azabu University, Sagamihara, Kanagawa, Japan; c Den-en-chofu Animal Hospital, Ota-ku, Tokyo, Japan; d Research and Education Center for Prevention of Global Infectious Diseases of Animals, Tokyo University of Agriculture and Technology, Fuchu, Tokyo, Japan; e Graduate School of Agriculture, Tokyo University of Agriculture and Technology, Fuchu, Tokyo, Japan; f Field Science Center, Tokyo University of Agriculture and Technology, Fuchu, Tokyo, Japan; g Research Institute of Biosciences, Azabu University, Sagamihara, Kanagawa, Japan; DOE Joint Genome Institute

## Abstract

This study reports the complete genome sequence of an African pygmy hedgehog adenovirus-1 isolate from an African pygmy hedgehog which displayed respiratory symptoms that included nasal discharge, sniffling, coughing, and respiratory distress. The viral genome is 31,764 bp long and shows four deletion sites compared to that of skunk adenovirus-1.

## ANNOUNCEMENT

Adenoviruses are nonenveloped, icosahedral viruses that range in diameter from 70 to 100 nm. Their double-stranded DNA genomes are linear and nonsegmented, range in size from 26 to 48 kb, and typically contain between 22 and 40 genes (1). Adenoviruses are subdivided into five genera, namely, Mastadenovirus, Aviadenovirus, Atadenovirus, Siadenovirus, and Ichtadenovirus. To our knowledge, over 44 species have been identified in the family *Adenoviridae*, and there is a wide range of host species capable of sustaining these viruses (2–5). Members belonging to the genus *Mastadenovirus* infect mammals. Skunk adenovirus-1 (SkAdV-1) is a recently described mastadenovirus isolated from a dead wild skunk (Mephitis mephitis) suffering from acute hepatitis in Canada (6). We previously reported the detection of an adenovirus possessing high homology with SkAdV-1 isolated from an African pygmy hedgehog (APH; Atelerix albiventris) suffering from tracheitis in Japan (7).

Here, we report the complete genome sequence of an African pygmy hedgehog adenovirus-1 (AhAdV-1) isolate, which was obtained from an African pygmy hedgehog with respiratory symptoms. A throat/nasal swab sample was collected from the African pygmy hedgehog, which was bred in-house in Japan from parents of unknown origin. The virus was isolated on a monolayer of MDCK cells cultured in Dulbecco’s modified Eagle medium (DMEM) containing 2% fetal calf serum and incubated at 37°C under a 5% CO_2_ atmosphere. The cell suspension was sonicated, and the debris of the cells was discarded. The virus suspension was supplemented with polyethylene glycol and NaCl, and the virus was purified by CsCl density gradient centrifugation (40,000 × *g*, 4°C for 2 h), as described by Nasukawa et al. (8). DNA extraction from the concentrated virus particles was carried out using a High Pure viral nucleic acid kit (Roche, Mannheim, Germany).

Deep sequencing was performed using a MiSeq benchtop sequencer (Illumina, San Diego, CA, USA). The MiSeq library was prepared with a Nextera XT DNA library prep kit (Illumina) and was sequenced using the MiSeq reagent kit v3 (600 cycles; Illumina) with 300 paired-end reads. Fastq files created by the MiSeq Reporter (Illumina) were imported into CLC Genomics Workbench 6.5.1 (CLC bio, Aarhus, Denmark). The MiSeq sequencing system produced a total of 2,326,998 reads (DDBJ accession no. DRA008804). The reads were trimmed using the quality control tool of the CLC Genomics Workbench, with default parameters. Twelve contigs containing one long contig (31,563 bp) were obtained using the *de novo* assembly tool of CLC Genomics Workbench using trimmed reads, with default parameters. The long contig was compared to skunk adenovirus PB1 (GenBank accession no. KP238322; 31,848 bp), and we found gaps in the genome and a lack of both ends of the genome. To obtain further information regarding the ends and gaps of the genome, a mapping consensus was obtained by mapping the reads to skunk adenovirus PB1, with default parameters. The long contig and mapping consensus of 2,303,932 reads were aligned to skunk adenovirus PB1, and we confirmed four deletions compared with SkAdV-1. The findings of both ends of the genome and the deletion sites were confirmed by PCR. We obtained a whole-genome sequence of the AhAdV-1 isolate (total, 31,764 nucleotides; 49% GC content). Previously, we detected adenovirus from an African pygmy hedgehog and reported this as SkAdV-1 (7). The whole-genome sequence determination revealed that there are some differences between the sequences of SkAdV-1 and this adenovirus (AhAdV-1). We registered this adenovirus isolate obtained from an African pygmy hedgehog as African pygmy hedgehog adenovirus-1.

### Data availability.

The complete genome sequence of the African pygmy hedgehog adenovirus-1 has been deposited to GenBank under the accession no. MK937781 and to the DDBJ under the accession no. DRA008804.
